# Evaluation of a pregnancy loss education intervention for undergraduate nursing students in Northern Ireland: A pre- and post-test study

**DOI:** 10.1186/s12912-023-01408-4

**Published:** 2023-08-14

**Authors:** Martina Galeotti, Suzanne Heaney, Martin Robinson, Áine Aventin

**Affiliations:** 1 School of Nursing and Midwifery, Queen’s University Belfast, Belfast, Northern Ireland United Kingdom; 2 Stress, Trauma and Related Conditions Research Centre, School of Psychology, Queen’s University Belfast, Belfast, Northern Ireland United Kingdom

**Keywords:** Pregnancy loss, Miscarriage, Termination of pregnancy, Abortion, Fetal anomaly, Parent, Experiences, Nurses, Nursing

## Abstract

**Background:**

Research highlights the importance of compassionate communication, adequate delivery of information, and professional support to help alleviate parental distress following pregnancy loss. However, many healthcare professionals do not feel sufficiently trained to deal with pregnancy loss in practice. We aimed to address this deficiency with an evidence-informed educational intervention to increase knowledge, skills, self-awareness, and confidence regarding pregnancy loss among UK nursing students.

**Methods:**

Educational resources, which included an 82-minute podcast and 40-minute online lecture were developed. The podcast focused on the lived experiences of three women who had experienced miscarriage, stillbirth, and termination of pregnancy for medical reasons. The pre-recorded lecture included definitions of types of pregnancy loss, discussion of the importance of communication, and information on the clinical management of pregnancy loss. Students were presented with both the lecture and podcast as a self-directed element of existing curricula. A pre-test/post-test cross-sectional survey design was used to investigate the impact of the educational intervention. The Perinatal Bereavement Care Confidence Scale (PBCCS) was completed by 244 first year BSc Nursing students before and up to a week after receiving the intervention. Quantitative data were analysed using a Paired Samples Wilcoxon test. Responses to open-ended questions, which allowed students to give feedback on the intervention content and delivery were analysed using Qualitative Content Analysis.

**Results:**

96% (n = 235) of the sample reported having no prior experience or training in the management and support of those experiencing pregnancy loss. At pre-test, 88% (n = 215) of students rated themselves as not confident in dealing with pregnancy loss in a professional capacity. Post-test, we found statistically significant effects for perceived competency on all learning outcomes (p < .001). Qualitative analysis of n = 745 individual text responses to open-ended questions indicated four categories related to the perceived value of using real-life stories for learning, demystifying a taboo subject, and providing tools for practice. Respondents suggested the inclusion of more information on memory-making, support networks, and mental health following pregnancy loss.

**Conclusions:**

The educational intervention increased student nurses’ perceived knowledge, confidence, and skills in caring for families experiencing pregnancy loss. This offers potential for increased quality of care for those experiencing pregnancy loss in healthcare settings, increased patient satisfaction, and improved mental health-related outcomes.

## Background

Perinatal loss is an umbrella term that refers to any pregnancy loss that occurs between conception and the first 28 days after birth. It includes miscarriage, ectopic pregnancy, stillbirth, neonatal death and termination of pregnancy for medical reasons (TFMR) [1]. Pregnancy loss refers to the intrauterine death of a fetus and therefore does not include neonatal death which occurs after birth. Globally, around 15% of pregnancies end in miscarriage [2] and around 2% end due to ectopic pregnancy, which is the implantation of the zygote outside the uterus [3]. The incidence of stillbirth is 2.9 per 1000 births in Western Europe [4, 5] and 13.9 per 1000 births worldwide [6]. European figures suggest that the TFMR prevalence rate is 4.6 per 1,000 births [7]. In the UK, over 70% of congenital anomalies are detected during pregnancy and, of those, around 37% will result in TFMR [8].

Pregnancy loss is an emotional and distressing event for expectant parents [9]; it is traumatic for many and can result in the development of long- and short- term psychological difficulties such as depression, anxiety, and post-traumatic stress disorder [10–12]. Further, women commonly describe feeling a sense of despair, failure and intense sadness following their loss [13, 14].

Many women and their partners report that their hospital experience played a key role in their emotional journey of pregnancy loss [15, 16]. As well as providing support for loss, research has indicated that a couples’ emotional wellbeing can be aggravated by their interactions with healthcare professionals (HCPs) [16, 17]. Studies report that HCPs can show a lack of understanding of the significance of pregnancy loss for women [17, 18]. Further, a lack of empathy and compassion has been observed in the interactions [15, 16]. It is acknowledged that caring for bereaved parents through loss is challenging for staff [19]. Healthcare professionals often report lacking confidence in their ability to provide good quality bereavement care for parents, describing it as emotional and distressing work [20, 21]. Furthermore, HCPs also highlight inadequate knowledge and training as a barrier to providing effective care [19, 21].

Previous studies based in USA, France, Ireland and Czech Republic investigated the effectiveness of educational training on pregnancy loss for HCPs [22–25]. Two studies evaluated the effectiveness of interventions targeting doctors and other HCPs to improve couple’s experience of pregnancy loss in hospital settings [23, 25]. These studies reported improved patient experience after the delivery of staff communication training. Two other studies reported positive effects from a series of workshops on compassionate communication in pregnancy loss for senior trainees in obstetrics and student midwives [23, 26, 27]. One study reported effectiveness of a 10-week series of e-learning materials on pregnancy loss, including lived experiences designed for nursing and midwifery students [26]. A 2021 scoping review of international literature [28] found 18 studies which examined interventions for preparing nurses and midwives for perinatal bereavement care, only two of which were conducted with UK nursing students. The review authors recommended further evidence-based resources for nurses and other HCPs which communicate bereaved parents’ psychological needs using e-learning strategies.

Nurses in the UK, and elsewhere, are likely to care for women experiencing pregnancy loss in several different clinical settings including emergency departments, genecology wards, surgical wards, and community settings. These encounters can occur during undergraduate clinical placements and beyond. Preparing student nurses for bereavement care is likely to positively impact the overall care women and partners receive in hospital settings, potentially ameliorating their physical and emotional wellbeing. This study aimed to evaluate the impact of a short educational intervention on pregnancy loss to first year nursing students.

## Methods

### Aim

This study aimed to determine the effectiveness of a short self-directed educational intervention in increasing nursing students’ self-reported knowledge, skills, awareness, and confidence regarding pregnancy loss.

### Design, setting and sample

A pre-test/post-test cross-sectional survey design was used to investigate the impact of the educational resources. The study was conducted with a convenience sample of first year pre-registration BSc Nursing students at Queen’s University Belfast (QUB), Northern Ireland. All 320 students enrolled in the ‘Evidence-based Nursing’ module who wished to take part in the study were eligible for inclusion. In order to maintain anonymity, we did not collect demographic information from participants. However, 94% of the cohort were female. Of the 320 students invited to complete the pre- and post-test questionnaires, 85.93% (n = 275) completed only the pre-test, while 76.25% (n = 244) completed at both timepoints and were therefore included in the analyses. At the time of the study, pregnancy loss education was not a part of the undergraduate curriculum.

### Intervention

Students received the educational intervention, which included a 40-minute online lecture and 82-minute podcast designed by MG, SH and ÁA and informed by findings from *The Bluebell Study* [29] and *The Miscarriage and Mental Health Study* [15], as well as other contemporary research [16]. Both studies included patient and public involvement and members of the public took part in the podcast. The students were assigned the materials as self-directed learning (i.e. they were provided with links to both resources and informed that viewing of both was mandatory). The podcast was facilitated by MG and SH with the participation of three women who shared their experience of miscarriage, stillbirth and TFMR. These three forms of pregnancy loss were included as they represent the most common forms of pregnancy loss students will encounter. The focus of the podcast was on care received in hospital and women’s perceptions of it. The pre-recorded lecture focused on different aspects of pregnancy loss and healthcare provision including communication and management. The lecture also included a short case study. Reflective of a change in UK guidelines regarding memory-making in perinatal bereavement care [30–33] and literature [34, 35] highlighting desired memory making among parents, we also included this topic within lecture. We also acknowledged that memory making may not be desired or beneficial for everyone, and the importance of choice [36]. A detailed overview of the content of the podcast and lecture can be found in Table 1.

**Table 1:** Content of the Educational Intervention

**Materials** 82-minute podcast and 40-minute lecture. Lecture content: **What is Pregnancy Loss?** -Types, prevalence, context in Northern Ireland **Impact of Pregnancy Loss on Parents** - Grief journey, impact on partners **Effective Communication and Pregnancy Loss** - Compassion and sensitivity, information needs of parents, responsive nursing care **Management of Pregnancy Loss in Practice** - Care and treatment options, clinical nursing care **Supporting parents to make memories** - Importance to parents, options and practical considerations personal account from a parent Podcast content: Three women talked about their lived experiences of miscarriage, still birth and TFMR. The experiences focused on the hospital care delivery and how this had impacted their journey of pregnancy loss, the grieving process, and recovery.
**Procedures** Students were able to access the lecture and podcast and listen to both at their own time in no specific order. Students were asked to complete a questionnaire before and after listening to the lecture and podcast.
**Content** The podcast and lecture content were developed and facilitated by a midwife, nurse, and psychologist. The podcast included 3 lived experiences (miscarriage, stillbirth, and termination of pregnancy due to fetal anomaly)
**Delivery** The podcast and lecture were provided to students via the educational platform ‘Canvas’ as part as their Evidence Based Nursing Module.
**Dose** All materials were available to students for a week. Students were able to complete the pre- and post-questionnaire within a week, specifically from the 10th to the 14th of October 2022.

### Recruitment

All first-year nursing students enrolled in the *Evidence-based Nursing* Module in October 2022 (n = 320) were contacted via email by their Year Lead, to inform them about the study. Participants received an information sheet about the study on the Module Announcements page (Canvas). The information sheet was also provided at the beginning of both questionnaires. The information sheet indicated to students their right to withdraw and that their responses would be anonymous. Students were provided with a link to complete a pre- and post-test questionnaire online using the secure software Qualtrics. The researchers were not involved in teaching the participants.

### Data collection

Descriptive information including previous experience of providing care in response to pregnancy loss and previous training in the area was collected. Overall, confidence with respect to care provision in response to pregnancy loss was rated on a scale from 1 ‘Not at all’ to 4 ‘Very’. Further, bespoke measurements of learning outcomes were collected (see Table 2) and, within the post-test questionnaire, 4 open-ended questions allowed feedback on the content and delivery of the educational materials (see below). An unlimited textbox was provided for responses. Responses to these questions were optional:

I. Which aspect(s) of the materials did you find most useful?
II. What topic(s) do you believe are needed to improve services in this area.
III. What improvements (if any) would you recommend to the materials?
IV. Any final reflections on the materials.

**Table 2:** Learning objective assessment questions

Please rate how competent you feel in relation to each activity:	
1	Ability to define types of pregnancy loss.
2	Ability to describe the impact of pregnancy loss on parents.
3	Ability to discuss the communication needs of parents experiencing pregnancy loss.
4	Ability to describe the clinical management of pregnancy loss.
5	Ability to discuss how to support parents to make memories.

Response Options: 1 (Not Competent), 2 (Somewhat Competent), 3 (Competent), 4 (Highly Competent)

The questionnaire also contained an adapted version of the Perinatal Bereavement Care Confidence Scale (PBCCS) [37]. The PBCCS includes 4 subscales: Bereavement Support Knowledge Scale; Bereavement Support Skills Scale; Self-Awareness Scale; and Organisational Support Scale. Scores are collated using a 5-point Likert scale ranging from strongly disagree to strongly agree (1–5). The adapted version of the PBCCS used in this study omitted Subscale 4 (Organisational Support), as this was deemed inappropriate due to respondents’ career stage. The adapted scale is presented in *Additional File 1*.

### Ethics

This study received ethical approval from Queen’s University Belfast, Faculty of Medicine, Health and Life Sciences Research Ethics Committee (REF MHLS 22_130). The lecture and podcast materials were presented to learners as mandatory within standard teaching provision, however, participation in this study was entirely voluntary. All participants provided informed consent prior to completing both questionnaires.

### Data analysis

#### Quantitative data analysis

Quantitative analysis was carried out by MR. Within-subjects change (i.e. assessment of change in the individual participants at the two timepoints) was examined using the self-reported measures of competency and confidence detailed in the data collection section above. Total scores were computed for each domain of the PBCC under investigation, Knowledge, Skills, and Self-Awareness. Measures of learning objective competencies and overall confidence in care provision for those experiencing pregnancy loss were not transformed, i.e., they were assessed as ordinal variables with a scale of 1 to 4 (see Table 2). These data were found to be non-normally distributed, therefore, change was analysed using a Paired Samples Wilcoxon test, and change in mean response (ΔM) between time points assessed.

### Qualitative data analysis

Qualitative content analysis was carried out by SH and MG on the open text questionnaire responses. This analysis approach was used because of its flexibility and content-focused approach to coding and categorising textual information. The aim was to find trends and patterns and explore relationships and structures of discourse to seek an understanding of the phenomenon [38, 39]. We followed the process as described by Bengtsson [40] (Table 3). This was conducted a collaborative process, followed by regular discussions between the entire research team to examine emerging codes, connections, meanings and themes. Consensus between the team remained high throughout the process.

**Table 3:** Qualitative Content Analysis. (adapted from Bengtsson, 2016 [40])

**Stage 1: Decontextualisation** *Identifying meaning units*	The written text responses were uploaded to NVivo 12 and read through several times to help the researchers get a sense of the whole and become familiar with and immersed in the data. Units of meaning were then coded.
**Stage 2: Recontextualisation** *Compare with the original data*	Original text was re-read alongside list of codes to check if all aspects of content were covered in relation to the aim.
**Stage 3: Categorisation** *Identify homogeneous groups*	Groups of similar codes were condensed together. Codes were then categorised according to their meaning.
**Stage 4: Compilation** *Draw realistic conclusions*	A manifest analysis was utilised, using the words of the participants, thereby staying close to the original meanings and context.

## Results

### Competency learning outcomes

Of the 244 respondents, 96.3% (n = 235) reported having no prior experience or training in the management and support of those experiencing pregnancy loss. At baseline most of the sample rated themselves as ‘Not at All’ (47.1%, n = 115) or ‘A little’ (37.7%, n = 92) confident in dealing with the issue of pregnancy loss in a professional capacity. Details of participants previous experience and self-reported competencies are shown in Table 4 below. All respondents reported engaging with both lecture and podcast during the study time period.

**Table 4:** Confidence and Training at Baseline

	n (%)
Age (*M*, *SD*)	22 (6.5)
Before attending the lecture/ listening to the podcast how confident did you feel dealing with cases of pregnancy loss?	
Not at all	115 (47.1)
A little	92 (37.7)
Somewhat	32 (13.1)
Very	5 (2.0)
Before attending had you received any training on these topics?	
Yes, from my employer	1 (0.4)
Yes, from my degree programme	0 (0.0)
Yes, self-directed learning	8 (3.3)
No	235 (96.3)
Previous professional experience providing care (through placement or work) for individuals and families experiencing pregnancy loss	6 (2.5)
How frequently do you deal with pregnancy loss in your placement?	
Daily	0 (0.0)
Weekly	2 (0.9)
Monthly or less	4 (1.6)
How adequate do you feel your training previously received on this topic has been?	
Extremely inadequate	86 (35.2)
Somewhat inadequate	27 (11.1)
Neither adequate nor inadequate	118 (48.4)
Somewhat adequate	8 (3.3)
Extremely adequate	5 (2.0)

**Fig. 1:**
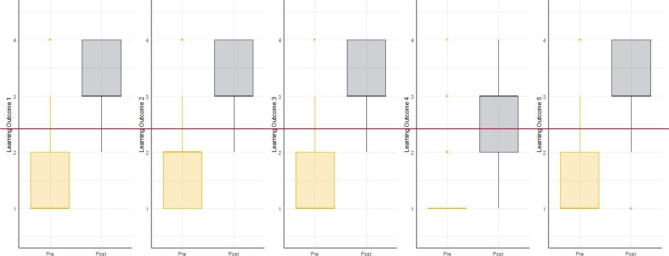
Change in Learning Outcomes Between Assessments

Changes in competency learning outcomes between pre- and post-test administration are shown in Fig. 1. These box and whisker plots show the distribution of responses at pre-test (yellow) and post-test (grey). The box depicts the inter-quartile range of responses on the 4-point Likert scale around the median shown in bold, while the full range in the data is plotted using the whisker lines and individual points for outliers (i.e. those outside the core distribution represented by the box and whisker).

Results of the Paired-Samples Wilcoxon tests showed that perceived competency was significantly increased for all competency learning outcomes (p < .001). Across all learning outcomes most participants rated feeling “*Not Competent*” or “*Somewhat Competent*” at baseline, and “*Competent*” or “*Highley Competent*” at post-test. Perceived competence in relation to Learning Objective 4 *“Ability to describe the clinical management of pregnancy loss*” at pre-test was however notably lower on average compared to the other objectives indicating this to be an area of perceived difficulty in this undergraduate learner group. A significant increase in perceived competency in this domain was found following participation, however, this remained low relative to the endorsement of other competencies post-test. Detailed numeric data for these results are provided in Table 5.

**Table 5:** Changes in Perceived Competency Learning Outcomes and Perinatal Bereavement Care Confidence Domains Between Pre- and Post-test Assessment

		Pre-Test		Post-Test			Wilcoxon	
	Range	M	SD		M	SD		V, sig.
Learning Outcome 1: Ability to define types of pregnancy loss	1–4	1.602	0.716		3.283	0.653		25,162***
Learning Outcome 2: Ability to describe the impact of pregnancy loss on parents	1–4	1.811	0.789		3.352	0.608		24,658***
Learning Outcome 3: Ability to discuss the communication needs of parents experiencing pregnancy loss	1–4	1.500	0.723		3.299	0.626		26,266***
Learning Outcome 4: Ability to describe the clinical management of pregnancy loss	1–4	1.197	0.491		2.906	0.705		27,122***
Learning Outcome 5: Ability to discuss how to support parents to make memories	1–4	1.603	0.716		3.283	0.653		24,921***
Bereavement Support Knowledge	15–75	50.119	5.656		58.725	3.986		28,479***
Bereavement Support Skills	9–45	24.586	5.635		33.959	4.034		28,551***
Self-Awareness	8–40	24.791	5.670		33.701	3.553		28,298***
Overall Confidence dealing with cases of pregnancy loss	1–4	1.643	0.737		3.258	0.624		25,433***

*** p < .001

### Perinatal Bereavement Care confidence

**Fig. 2:**
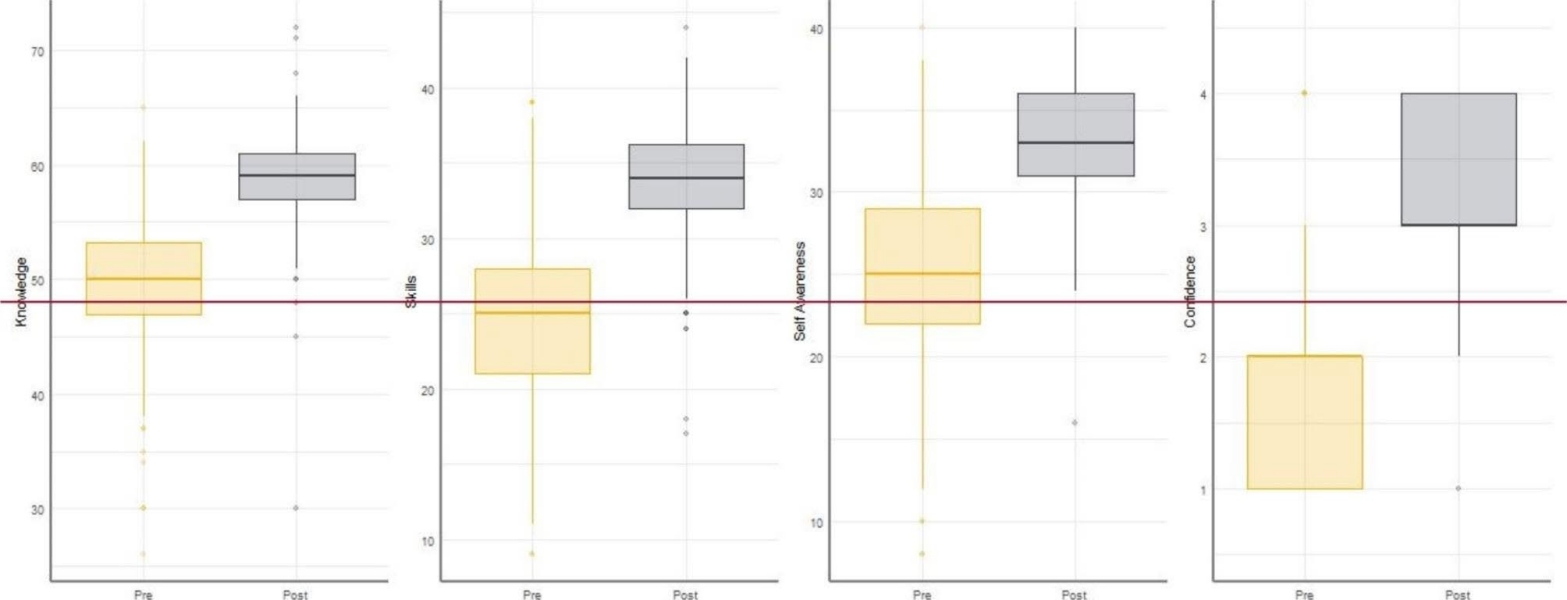
Change in PBCC Subdomain Measures (Bereavement Care Knowledge, Skills, Self-Awareness) and Self Rated Confidence Between Assessments

Changes between pre- and post-test measures of perceived confidence in providing perinatal bereavement care were estimated using mean response on the PBCC subscales and overall confidence rated from 1 to 4 (see Fig. 2).

There was a statistically significant increase (V = 28,479, p < .001) in mean reported knowledge following participation relative to baseline (ΔM = + 8.61). Likewise, participants reported a statistically significant increase (V = 28,551, p < .001) in perceived skills at post-test relative to baseline (ΔM = + 9.37). Participants similarly reported a statistically significant increase (V = 28,298, p < .001) in perceived self-awareness around perinatal loss at post-test relative to baseline (ΔM = + 8.91). Finally, there was a statistically significant increase (V = 25,433, p < .001) in perceived overall confidence in providing professional care for those experiencing perinatal/pregnancy loss at post-test relative to baseline (ΔM = + 1.61).

### Analysis of qualitative comments

**Fig. 3:**
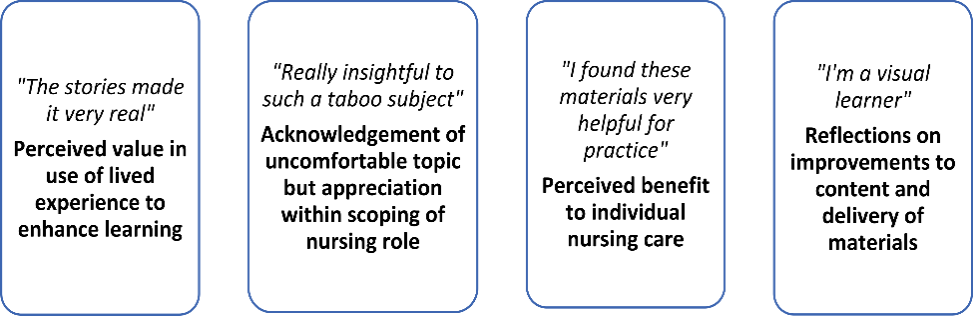
Categories identified by analysis of qualitative responses relating to participant perceptions of the intervention

A total of 745 individual text responses were received across the four optional open-ended questions. Owing to the qualitative nature of our analysis approach and focus on understanding and interpreting nursing students’ perceptions of the intervention, no quantifiable counting or measurements of data are reported. Analysis of student responses identified four categories (Fig. 3).

### “The stories made it very real” - Perceived value in use of lived experience to enhance learning

Many comments indicated the perceived value and impact of hearing the lived experience of the women who took part, which included three women in the podcast and one case study in the lecture. While many stated it was emotive and, for some, difficult to listen to the stories, there was consensus that they facilitated a greater understanding of the parents’ experience and perception of their healthcare needs. Several noted that they would have liked to hear a male or partners’ perspective to understand their particular experience and needs more fully.

*“I found it useful hearing real life examples which has taught me to be very careful with what I say and when I say it”.*

*“I would be interested in hearing from the male point of view about how he felt during the miscarriage”.*

### “Really insightful to such a taboo subject” - Acknowledgement of uncomfortable topic but appreciation within scoping of nursing role

Several responses referred to the uncomfortable nature of the topic pregnancy loss, which was described as *“taboo”, “heart-breaking”, “difficult”, “sensitive”* and associated with *“stigma”.* However, there was consonance about the need to cover this topic and not *“shy away from it”*, as well as gratitude, *“thank you for the resources. They are really helpful, and I will definitely revisit these and incorporate it into my learning”.*

*“It was very emotional to hear but also extremely important, so students are somewhat prepared to know what to do and say in these situations as well as know what not to say or do”.*

### “I found these materials very helpful for practice” - Perceived benefit to individual nursing care

Findings identified what respondents felt was important to parents through their pregnancy loss care experience and they reflected on how they would take this forward in their nursing practice. Several referenced upcoming clinical placements in which they felt this learning would be useful, while others reflected on its potential use across their career.

*“I will carry these materials with me throughout my career”.*

*“As a male, I find we have very little teaching toward pregnancy issues and this resource was great to learn from”.*

While a small number of students disclosed a personal experience related to the topic, both direct and indirect, most students expressed little prior knowledge or experience surrounding pregnancy loss. Increased knowledge was identified as one of the main benefits from taking part in this education. Specific mention was given to understanding different types of pregnancy loss and the clinical management of pregnancy loss.

*“This resource provided me with a greater depth of knowledge. Also listing the support providers who are available outside health care setting is a great resource”.*

### “I’m a visual learner” - Reflections on improvements to content and delivery of materials

Overall, feedback about the materials and content was positive. For those who commented on the length of the materials, most stated that they felt the podcast should be shorter, or that it should be split into smaller sections to maintain engagement. Smaller sections were also suggested *“as the content is very heavy”.* While most were satisfied with the podcast and lecture format, feedback for improvement centred around increasing the visual content and several also stated a wish for a more interactive elements to the learning materials. Suggestions included, videoed examples of effective care, roleplays in class, and an interactive workshop.

*“I would maybe include a video for the visual learners around the topic of baby loss”.*

*“Shorter but more frequent podcasts”.*

*“Introduce more student interaction”.*

*“Role play scenarios for class or a quiz at the end to test self-learning”.*

*“Videos of health professionals communication”.*

Students reported positively on the content of materials and there was a clear desire for further learning in this area. A variety of responses were received relating to desired future content. As noted, some respondents would like to learn about the male or partner experience of pregnancy loss, and several wished for information on the impact of pregnancy loss on other family members, such as grandparents and siblings and how best to support them. Others wished for practical information such as how to do memory making with parents, more information on support services they could signpost parents to or developing communication skills in difficult circumstances.

*“More attention on how as nurses we could make more memories for the parents”.*

*“Further provision and awareness of the support networks that are available to women and partners after the experience of pregnancy loss”.*

*“More awareness of the long-term impact on mental health from pregnancy loss”.*

## Discussion

This study presents novel findings on the effectiveness of a short, self-directed, educational intervention for improving UK nursing students’ perceived knowledge, skills and confidence around the topic of pregnancy loss. Echoing the results of previous research [23, 26] the findings indicated that the intervention resulted in statistically significant increases in students’ perceived ability to define types of pregnancy loss, describe its impact on parents, discuss the communication needs of parents’ needs, describe the clinical management of pregnancy loss, and discuss how to support parents to make memories after their loss.

### Implications for nursing education in pregnancy loss

In line with other research, students who took part in this study reported limited exposure to pregnancy loss during clinical and university training [28]. Further, our findings support those of previous studies which highlighted that most HPCs do not feel adequately trained on the topic [29, 41–43]. This suggests an urgent need for the development and implementation of educational programmes for nurses, who are likely to care for patients experiencing pregnancy loss in emergency rooms, gynaecology and surgical wards and in community settings [43]. A recent review [28] highlights international guidelines and standards that might inform such curricula. In the UK, this work could be informed by UK Nursing & Midwifery Council practice standards for registered midwives [44, 45], which require proficiency in perinatal bereavement care – requirements which are notably absent from current proficiency standards for registered nurses [45]. Further research should explore the impacts of including this intervention in the curriculum and also providing such education for nurses in practice.

The findings of this study also suggest promising content for pregnancy loss educational interventions. Adding to findings of other research [26, 42, 43], the need for content to increase knowledge of the different types of pregnancy loss and related clinical management strategies, as well as the physical and emotional impact on parents and their communication needs, were highlighted. Findings also indicated the value of using the lived experiences of bereaved parents to enhance learning. While students appreciated the mix of modalities used in the current intervention, the use of more skills-based, interactive approaches was recommended, particularly around confidence communicating with parents. Previous studies which have highlighted key lessons in developing communication skills in pregnancy loss among health practitioners, will be informative in this regard [46–49].

Further recommendations for educational intervention development suggested by participants included the need for understanding of the needs of fathers/partners and the wider family, as well as practical information on how to use tools such as memory making, and more information on available support services for parents. In the UK, the National Bereavement Care Pathway for Pregnancy and Baby Loss [48] recommends bereavement care training for all professionals who work with bereaved parents and provides detailed toolkits that could be implemented in education and practice.

Finally, the findings recognise the potential emotional and psychological impact of learning about and working with bereaved parents on nurses. Students who listened to the lived experiences of bereaved parents, reported feeling an emotional toll. As recommended by other research [26, 50], educational interventions on pregnancy loss should aim to increase self-awareness and sources of further support for student nurses.

### Strengths & limitations

The strengths of this study lie in its use of a novel educational intervention and reporting of findings from a sample of undergraduate UK nursing students. It should be noted that the outcome measure of interest in this study centres exclusively on self-report ratings of perceived competence and confidence. Likewise, the post-test measurement was obtained in quick succession after engaging with the lecture and workshop. These results should therefore be interpreted cautiously as the longevity of this change remains unknown. It is possible that measurement of confidence and competence in the abstract and in such a short timeframe lend themselves to inflated perception of efficacy. Future investigations may consider assessment of confidence and behaviour change over a longer latency period including time spent in clinical practice to better measure the effectiveness of brief educational intervention in this context. Further research should consider the development of evaluations informed by relevant international guidelines and the use of robust evaluation methods, such randomised controlled trials, or quasi-experimental studies.

## Conclusion

This study demonstrated that a brief, self-directed, educational intervention increased perceived knowledge, skills, and confidence in providing professional support in the context of pregnancy loss. Students positive feedback stressed the importance of introducing this as part of university curricula as it highlighted many benefits, not only increasing their perceived competency, but also promoting self-reflection on current practice and parents’ and wider family needs. The study highlighted key implications for further development and evaluation of pregnancy loss educational interventions, including the importance of including interactive educational modalities such as communication skills practice, and practical skills in implementing memory-making activities with parents. The findings also highlighted the importance the need to understand the emotional and psychological implications of pregnancy loss bereavement for both parents and practitioner nurses.

## Data Availability

All data resulting from the research in this study will be stored for a minimum of 5 years and a maximum of 10 years in Queen’s University Belfast. All data will be archived by year 10 in The UK Data Archive (UKDA) located in the University of Essex. This is a centre that specialises in keeping data securely so that other researchers can apply for ethical permission to use it in their own research. Intervention materials are freely available upon request to the corresponding author.
